# Supplementary material for: Potential prognostic factors for hamstring muscle injury in elite male soccer players: A prospective study
Source: PLoS One. 2020 Nov 9;15(11):e0241127. doi: 10.1371/journal.pone.0241127 (PMC7652257; doi:10.1371/journal.pone.0241127)
Supplement: Attachment — Submitted filename: Response to Reviewers.docx [file pone.0241127.s003.docx]

Comments to the Author

The authors would like to thank the Editor and Reviewer 2 for his/her positive and thought-provoking comments, which have improved the overall quality of the paper. The manuscript has been changed based on the reviewer’s comments and our point-by-point responses, highlighting the changes made are outlined below. In the text of the revised manuscript the color highlights the modified sentences.

5. Is the manuscript presented in an intelligible fashion and written in standard English?

PLOS ONE does not copyedit accepted manuscripts, so the language in submitted articles must be clear, correct, and unambiguous. Any typographical or grammatical errors should be corrected at revision, so please note any specific errors here.

The authors thank the Editor and Reviewer 2 for their request to further improve the English language in the manuscript and the corrections were made accordingly.

6. Review Comments to the Author

Please use the space provided to explain your answers to the questions above. You may also include additional comments for the author, including concerns about dual publication, research ethics, or publication ethics. (Please upload your review as an attachment if it exceeds 20,000 characters)

Reviewer #2: The authors provided reasonable changes according with the reviewers opinion. Despite that I still maintain my reserves about the novelty of your study. In this sense I just have minor suggestions.
- At the end you should add a limitations' section to better describe the flaus of the intervention;
- Please make your conclusions more clearly for the reader. If the Nordic exercise performance predicts better the hamstrings injury incidence, this should be one of your key points.

The authors thank Reviewer 2 for his/her valuable comments concerning the limitations of the study and the respective changes can be found in: page 21; lines 448-455.

Despite a novel and comprehensive methodology including a homogenous study population, and a vast number of field and laboratory tests, some potential limitations are worth noting. Notably, a longer monitoring period could provide a better prediction depiction of HSIs. Further, different, diagnostic, prevention, and rehabilitation protocols implemented by the teams’ staff could have influenced the results. Therefore, further research including standardized diagnostic and preventive, and load monitoring measures are of utmost importance in order to better predict HSIs in soccer players.

The authors thank Reviewer 2 for his/her valuable comments with respect to the conclusions section and have improved the section as follows: page 21-22; lines 462-463.

Age, NHST, previous injury history, and concentric hamstring strength at 240°/sec resulted to be the best prediction factors of HSIs.
